# How nursing practice environments limit implicit rationing of care and nurse-assessed adverse events: the role of flow at work

**DOI:** 10.1186/s12912-023-01644-8

**Published:** 2024-01-03

**Authors:** Heba E. El-Gazar, Ali D Abousoliman, Mona Shawer, Paulo Coelho, Mohamed A. Zoromba

**Affiliations:** 1 Nursing Administration Department, Faculty of Nursing, Port Said University, Port Said, Egypt; 2 Nursing Department, College of Applied Medical Sciences, Prince Sattam bin Abdulaziz University, Al-Kharj, Saudi Arabia; 3 Nursing Administration Department, Faculty of Nursing, Kafrelsheikh University, Kafr el-sheikh, Egypt; 4 High Institution of Nursing, Mansoura, Egypt; 5 Nursing Department, Prince Sultan Cardiac Center, Riyadh, Saudi Arabia; 6 Psychiatric and Mental Health Nursing Department, Faculty of Nursing, Mansoura University, Mansoura, Egypt; 7 Nursing Education and Training, King’s College Hospital London, Jeddah, Saudi Arabia

**Keywords:** Adverse events, Flow at work, Nurses, Nursing practice environment, Rationed care

## Abstract

**Background:**

The nursing practice environment is beneficial in curbing implicit rationing of nursing care and adverse patient events. However, the underlying mechanisms of these relationships remain unexplored.

**Aim:**

To test whether flow at work mediates the relationship between the nursing practice environment, implicit rationing of nursing care, and nurse-assessed adverse patient events.

**Methods:**

This cross-sectional study involved 231 nurses from five hospitals in Port Said, Egypt. The participants completed Arabic-translated versions of the Practice Environment Scale of the Nursing Work Index, the Work-Related Flow Inventory, the Perceived Implicit Rationing of Nursing Care instrument, and the Adverse Patient Events scale. Structural equation modeling was used to test the hypothetical model.

**Results:**

The favorable nursing practice environment positively predicted nurses’ flow at work (β = 0.64, *p* < 0.001), while inversely predicting implicit rationing of nursing care (β = -0.23, *p* = 0.014) and adverse patient events (β = -0.35, *p* < 0.001). Nurses’ flow at work inversely predicted implicit rationing of nursing care (β = -0.30, *p* = 0.002) and adverse patient events (β = -0.29, *p* = 0.002). Moreover, nurses’ flow at work acted as a mediator, linking the nursing practice environment to the rationing of nursing care and adverse patient events, with 500 bootstrap results for the indirect effects (β = -0.24, *p* = 0.001, 95% CI: -0.43 to -0.09; and β = -0.44, *p* = 0.003, 95% CI: -0.79 to -0.16, respectively).

**Conclusion:**

Nurses working in a favorable nursing practice environment are more likely to experience flow at work, limiting implicit rationing of nursing care and adverse patient events.

**Implications for nursing management:**

Nursing administrators should strive to create a healthy nursing practice environment to foster nurses’ flow and thereby reduce the frequency of implicit rationing of nursing care and adverse patient events.

**Supplementary Information:**

The online version contains supplementary material available at 10.1186/s12912-023-01644-8.

## Introduction

Patient safety and the provision of high-quality nursing care are ongoing concerns among nursing scholars and practitioners globally. They represent salient issues in many healthcare settings [1]. The most commonly used indicators to measure the safety and quality of care provided are implicit rationing of nursing care (IRNC; the omission or inability to perform essential nursing tasks) and adverse patient events (APE; incidents that lead to unintended harm to patients) [2]. IRNC and APE have significant negative patient outcomes, including poor patient satisfaction, increased length of stay, patient safety violations, high morbidity, and mortality rates, and patient readmission [3].

Given these negative outcomes, it is essential to limit IRNC and APE in healthcare settings [2]. Hence, scholars call for studies to explore factors that may act against such phenomena [3, 4]. Prior research has focused on studying organizational factors as triggers of IRNC and APE, such as inadequate staffing levels [1], leadership styles [5, 6], workload [7], nurses’ breaks [8], and work environment [9, 10], but to our knowledge, nurse-individual factors (i.e., flow at work) have been significantly overlooked.

Experiencing flow at work is an essential prerequisite for well-being and functioning [11]. However, there is scant research on flow among nurses. As an exception, Zito et al. studies have shown the role of nurses’ flow in decreasing work exhaustion [12, 13], and the study by Martínez-Zaragoza et al. affirmed that nurses’ flow could enhance their health [14]. This study attempts to overcome this shortcoming by exploring the link between nurses’ flow at work and IRNC and APE.

Although previous studies have indicated the role of a positive workplace environment in curbing IRNC and APE, the mechanism explaining how the nursing practice environment is linked to IRNC and APE represents a research gap [10, 15]. This study seeks to fill this gap by drawing on flow theory [16] and Donabedian’s structure-process-outcome framework [17], proposing that flow at work may act as an intervening mechanism in linking the nursing practice environment to IRNC and APE. Therefore, this study aims to test whether flow at work mediates the relationship between the nursing practice environment, IRNC, and nurse-assessed APE.

## Literature review and hypotheses development

### Nursing practice environment

The nursing practice environment refers to the work conditions that assist or hinder professional nursing activities. The benefits of such environments are well-documented in nursing literature, including improved nurses’ sense of coherence [18], job satisfaction, interprofessional collaboration, work effectiveness, and patient safety outcomes [10].

### Hypothesis 1: nursing practice Environment and Flow at Work

Flow at work represents an optimal state of mindfulness where individuals are fully engaged and highly conscious of their work activities [16]. According to flow theory, experiencing flow in the workplace is influenced by the active interaction between employees and their environment. It is also affected by their subjective perception of the work environment [16]. In a healthy nursing practice environment, nurses have access to supportive leadership, adequate staffing levels, and respectful relationships among colleagues, all of which contribute to nurses’ positive perceptions of their work environment [19]. Moreover, research has affirmed that job resources are essential to nurses’ flow [12]. A healthy nursing practice environment provides physical, mental, and social resources for nurses [20]. Therefore, we propose the following hypothesis:

*H1. A favorable nursing practice environment is positively correlated with nurses’ flow at work*

### Hypothesis 2 and 3: nursing practice environment, IRNC, and APE

IRNC is defined as any element of nursing care required but not performed or postponed [21]. Limiting IRNC is critical for directly curbing adverse patient events (APE), such as medication errors, patient falls, and hospital-acquired infections [1, 3]. Current research suggests that the nursing work environment may decrease IRNC and APE [9, 10]. Such links could be explained using the Person-Environment-Occupation Performance (PEOP) model, which signifies the role of environmental factors, such as the work environment, in enhancing employee performance [22]. In the nursing context, good performance is reflected in providing comprehensive care with no missed care (i.e., IRNC) or patient incidents (i.e., APE) [1]. Therefore, we propose the following hypotheses:

*H2. A favorable nursing practice environment is inversely correlated with the IRNC.*

*H3. A favorable nursing practice environment is inversely correlated with the APE.*

### Hypothesis 4 and 5: flow at work, IRNC, and APE

According to Csikszentmihalyi, flow is a condition of being deeply involved and absorbed in a task with total concentration [16]. Individuals in flow are not distracted, have higher engrossment, utilize their skills and energies to the utmost, and are completely focused on their activities [11]. This enables them to perform optimally [23]. Applying this concept to nursing, it is reasonable to argue that such a state may enhance nurses’ ability to provide comprehensive care and remain alert to potential patient incidents. Therefore, we propose the following hypotheses:

*H4. Nurses’ flow at work is inversely correlated with IRNC.*

*H5. Nurses’ flow at work is inversely correlated with APE.*

### Hypothesis 6 and 7: flow at work as a mediator

Drawing on Donabedian’s structure-process-outcome framework, enhancing the hospital’s structure can improve the work process, leading to better outcomes [17]. In this study, structure represents the nursing practice environment, nurses’ flow represents the work process, and the outcomes include IRNC and APE. Previous studies have also investigated the nursing practice environment as a structural factor [24], flow as the main process for effective performance [23], and IRNC and APE as outcomes [2]. Therefore, we propose the following hypotheses:

*H6. Nurses’ flow at work mediates the correlation between nursing practice and IRNC.*

*H7. Nurses’ flow at work mediates the correlation between the nursing practice environment and APE.*

**Fig. 1:**
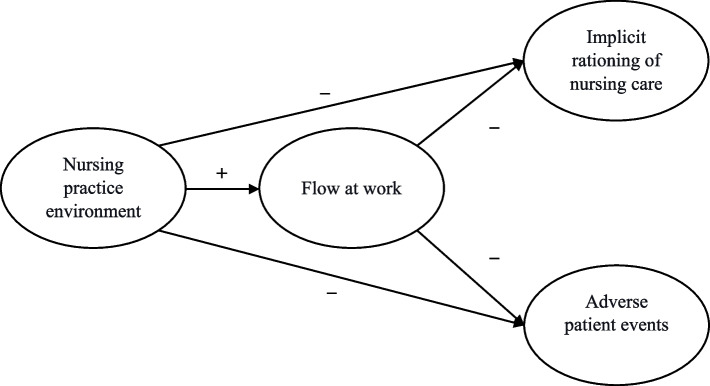
The hypothetical study model

Figure 1 shows the hypothetical study model.

### Methods

#### Study design

A descriptive cross-sectional study investigated whether flow at work mediates the relationship between the nursing practice environment, IRNC, and nurse-assessed APE. The study adhered to the STROBE statement, which provides guidelines for reporting observational studies.

#### Participants

The study involved clinical nurses in oncology, medical, surgical, or intensive care units across five Universal Health Insurance hospitals in Port Said, Egypt. The sample size was calculated using power analysis for the structural equation modeling (SEM) method, as proposed by a previous study [25]. This method uses the degrees of freedom (df) for the tested model and the subsequent equation:

$$df=p(p + 1)/2-\text{q} = 19(19 + 1)/2-43 = 147$$

Where p is the number of observed variances (*p* = 19) and q is the number of free parameters in the tested model (q = 43; 5 regression path + 15 factor loading + 22 error variance + 1 factor variance). With a power of 0.95 and 𝛼 = 0.01, a sample size of 226 nurses was required for this study model with df = 147 to achieve RMSEA = 0.05. To account for a 20% attrition rate, 271 nurses were recruited through convenience sampling.

Eligible nurses were licensed staff nurses who had worked in direct patient care for at least one year on a shift basis. Nurses were excluded if they were interns, had cognitive or psychological problems, or did not work continuously from October 1, 2021, to the data collection period due to maternity or sick leave. Of the 271 invited participants, 258 responded, of which 27 were not included in the analysis due to missing data. Thus, the final sample included 231 nurses, resulting in an 85.24% response rate.

### Instruments

Data were collected using a five-section questionnaire consisting of the Introductory Information Form, the Practice Environment Scale of the Nursing Work Index (PES-NWI), the WOrk-reLated Flow Inventory (WOLF), the Perceived Implicit Rationing of Nursing Care (PIRNCA) instrument, and the Adverse Patient Events (APE) scale.

As the measures were initially constructed in English, Arabic versions were created using the translation-back-translation protocol [26]. Initially, two English professors independently translated the scales from English to Arabic. Subsequently, these translators collaborated with the primary author to resolve any discrepancies and create an initial Arabic draft. This draft was then back-translated into English by a certified translator. Afterward, the authors, along with the three translators involved in both the forward and back translations, compared the translated and back-translated versions with the original scales to produce a consolidated Arabic version. Following this, a panel of five experts, which included two nursing professors, one nursing director, and two ward nurses with master’s degrees in nursing, reviewed the translated version alongside the original to ensure equivalence of terms and verify that the language was clear and straightforward. Some words were modified to better suit the context of Egyptian nurses. Finally, the Arabic version of the scales underwent pre-testing with 27 nurses to ensure its clarity, applicability, and comprehensibility before conducting the main study.

The Introductory Information Form included demographic questions asked participants about their age, gender, marital status, education, unit specialty, and years of experience in nursing and the present unit.

The PES-NWI [19] was used to operationalize the nursing practice environment. The PES-NWI contained 31 items grouped into five dimensions: participation in hospital affairs (9 items), nursing foundations for quality of care (10 items), nurse manager ability, leadership, and support of nurses (5 items), staffing and resource adequacy (4 items), and collegial nurse-physician relations (3 items). Responses were rated on a 4-point Likert scale (1 = completely disagree; 4 = completely agree). A higher score indicates a more favorable nursing practice environment. In the original study, Cronbach’s alpha for the five dimensions ranged from 0.71 to 0.84.

*The WOLF* [27] was used to gauge nurses’ flow levels at work. The WOLF contained 13 items grouped into three dimensions: absorption (4 items), work enjoyment (4 items), and intrinsic work motivation (5 items). Responses were rated on a 7-point Likert scale (1 = never; 7 = always). Higher scores indicate greater flow at work. In the original study, Cronbach’s alpha for the three dimensions ranged from 0.75 to 0.90.

The PIRNCA instrument [4] was used to gauge nursing care rationing. PIRNCA consisted of 31 items covering six nursing activities: assistance with physical care (8 items), the implementation of the prescribed treatment plans (6 items), emotional support and teaching (3 items), surveillance/vigilance (7 items), coordination of care and discharge planning (3 items), and documentation (4 items). Participants rated the frequency of each of the 31 nursing care activities not performed during the past seven working shifts using a 4-point Likert scale (1 = rarely; 4 = always). An additional “nonapplicable = 0” option was included for activities not assigned to a particular unit. The total mean score was estimated by computing the average scores for items answered between 1 and 4 and treating “nonapplicable” as missing values [28]. A higher score represents a higher level of implicit nursing care rationing. In the original study, Cronbach’s alpha was 0.97.

The APE Scale [29] was used to evaluate nurse-assessed adverse patient events. APE measured five negative patient events: (1) patient and family complaints, (2) verbal abuse, (3) patient falls, (4) hospital-related infections, and (5) medication administration errors. To capture an adequate account of these relatively infrequent events, nurses were asked to report the frequency of each event during their shifts over the past year using a 7-point Likert scale (0 = never; 6 = daily). A higher score represents a greater frequency of adverse patient events assessed by nurses. The APE Scale is a valid and reliable measure extensively employed in nursing research [6, 29–31]. In the original study, Cronbach’s alpha was 0.75.

### Pre-study

The study questionnaire was initially pretested with a convenience sample of 27 nurses not included in the main study. Subsequent to filling out the questionnaire, the nurses were interviewed to evaluate its clarity, applicability, and comprehensibility. The nurses confirmed that the questionnaire items were clear and easily understood. The average time to answer the questionnaire was 15 min. Furthermore, the psychometric properties were evaluated, affirming the validity and reliability of the Arabic-translated scales. In this pre-study phase, Cronbach’s alpha values were 0.94 for the PES-NWI, 0.92 for the WOLF, 0.93 for the PIRNCA instrument, and 0.87 for the APE scale.

### Data collection

Data were collected between October and December 2022. Firstly, the researchers sent an introductory letter and a copy of the study questionnaire to the managers of the five participating hospitals for review and approval. After gaining initial approval, the researchers contacted the head nurses of each unit to provide them with background about the study and facilitate the nurses’ recruitment and data collection processes. Upon getting the head nurses’ approval, potential participants were informed about the study and requested an informed consent. The researchers personally delivered the questionnaire to participants in their unit, enclosed in a brown envelope. The questionnaire commenced with a description of the study and assured confidentiality, voluntary participation, and the academic purpose of the study. Participants were instructed to seal the answered questionnaire in an envelope and send it back to the researchers within one week. Further, within this week, we remind nurses once to complete the questionnaire. Additionally, within this week, we reminded nurses once to complete the questionnaire.

### Statistical analysis

The data were analyzed using IBM SPSS version 28.0 and AMOS version 24.0. Variables were screened for missing values; cases with up to 10% missing data were imputed by mean scores, and cases exceeding 10% were deleted listwise [32]. Descriptive statistics were computed to summarize sample demographics and study variables. The correlation between the study variables was tested using Pearson’s correlation analysis. The validity and reliability of the study model were assessed. The proposed model was evaluated by SEM with 5000 bootstrapping resamples at a 95% confidence interval. The goodness-of-fit statistics were tested against the following parameters: the ratio of the chi-square to degrees of freedom (χ2/df) ≤ 3.00, the Incremental Fit Index (IFI), Tucker Lewis Index (TLI), the Comparative Fit Index (CFI) ≥ 0.90, and the root-mean-square error of approximation (RMSEA) ≤ 0.08 [33]. Statistical significance was specified at a 5% significance level.

## Results

### Sample demographics

Most participants were female (72.7%), with a mean age of 32.48 years (SD: 8.15). Of the participants, 48.9% were married, 36.3% held a bachelor’s degree in nursing, and 35.5% worked in an intensive care unit. On average, the participants had spent 11.90 years (SD: 7.91) in the nursing profession and 4.61 years (SD: 2.95) in their current working unit (Table 1).

**Table 1:** Sample demographics (*N* = 231)

Characteristic	Category	no	Percent	Mean (SD)	Range
Age (years)	< 30	97	42	32.48 (8.15)	20–55
	30–45	89	38.5		
	> 45	45	19.5		
Sex	Male	63	27.3		
	Female	168	72.7		
Marital status	Single	94	40.7		
	Married	113	48.9		
	Divorced	15	6.5		
	Widowed	9	3.9		
Education	Diploma	69	29.9		
	Associate	64	27.7		
	Bachelor	84	36.3		
	Postgraduate	14	6.1		
Unit	Medical	76	32.9		
	Surgical	46	19.9		
	Oncology	27	11.7		
	Intensive care	82	35.5		
Years in the profession	≤10	112	48.5	11.90 (7.91)	1–31
	> 10	119	51.5		
Years in the working unit	≤ 5	143	61.9	4.61 (2.95)	1–12
	> 5	88	38.1		

### Common method variance (CMV)

The study data were cross-sectional and self-reported, making them vulnerable to CMV. To ensure the data were free from contamination by CMV, we conducted a pre-study consultation with relevant experts to guarantee the clarity and comprehensibility of the scale items. Additionally, participants were assured in the cover letter that their names were not required to eliminate their suspicion of factually answering questionnaires [34]. Furthermore, we statistically examined CMV presence in the data using Harman’s single-factor test. Results showed that the single factor explained 32.18% of the variance, below the acceptable 50% value [35], indicating that the study data had no CMV risk.

### Measurement model

Confirmatory factor analysis (CFA) was performed to confirm the distinctness of the study constructs. As presented in Table 2, the four-factor model (i.e., nursing practice environment, flow at work, IRNC, and APE) had a better fit to the data (χ2 = 320.43, df = 146, χ2/df = 2.19, IFI = 0.95, TLI = 0.94, CFI = 0.95, RMSEA = 0.072) than all of the other models, confirming the distinctness of the four constructs.

**Table 2:** Measurement model (*N* = 231)

Model	χ2	df	χ2 /df	IFI	TLI	CFI	RMSEA
Hypothesized four-factor model	320.43	146	2.19	0.95	0.94	0.95	0.072
Three-factor model (NPE and FW combined)	448.57	149	3.01	0.91	0.89	0.91	0.093
Two-factor model (NPE and FW combined, IRNC and APE combined)	1154.58	151	7.65	0.69	0.65	0.69	0.170
One factor model (all combined)	1674.67	152	11.02	0.54	0.48	0.53	0.209

*APE* Adverse patient events, *FW* Flow at work, *IRNC* Implicit rationing of nursing care, *NPE* Nursing practice environment

### Validity and reliability

The validity of the translated scales was assessed in terms of content, convergent, and discriminant validity. To evaluate content validity, five nursing professors rated each item on a 4-point Likert scale, ranging from 1 (not relevant) to 4 (highly relevant). An item-level Content Validity Index (I-CVI) ≥ 0.78 and a scale-level CVI/average (S-CVI/Ave) ≥ 0.90 were considered satisfactory for content validity [36]. The I-CVI scores ranged from 0.95 to 1.00 for the PES-NWI, 0.97 to 1.00 for the WOLF, 0.95 to 1.00 for the PIRNCA instrument, and 0.96 to 1.00 for the APE scale. The S-CVI/Ave was 0.96 for the PES-NWI, 0.98 for the WOLF, 0.97 for the PIRNCA instrument, and 0.97 for the APE scale.

Convergent validity was checked, as recommended by Hair et al., through the following: (1) factor loadings, which are required to be significant with values above 0.5; (2) construct reliability (CR), which should be above 0.7; and (3) average variance extracted (AVE), which needs to be above 0.5 [32]. As presented in Table 3, all factor loadings were significant and ranged from 0.66 to 0.95. The values of CR ranged from 0.86 to 0.97, and the values of AVE ranged from 0.66 to 0.86, providing evidence of the convergent validity of the study variables.

**Table 3:** Descriptive statistics, measurement validation and correlations of studied variables (*N* = 231)

Variable	Mean (SD)	α	Factors loading	CR	AVE	MSV	1	2	3	4
1. NPE	2.67 (0.54)	0.95	0.66–0.94	0.91	0.67	0.41	**(0.82)**	*0.591*	*0.419*	*0.526*
2. FW	4.39 (1.14)	0.92	0.73–0.95	0.86	0.66	0.40	0.55***	**(0.81)**	*0.421*	*0.501*
3. IRNC	1.82 (0.77)	0.97	0.72–0.91	0.97	0.86	0.19	-0.38***	-0.39***	**(0.93)**	*0.400*
4. APE	2.89 (1.35)	0.91	0.74–0.89	0.92	0.68	0.28	-0.48***	-0.49***	0.37***	**(0.82)**

*APE* Adverse patient events, *AVE* Average variance extracted, *CR* Composite reliability, *SD* Standard deviation, *FW* Flow at work, *IRNC* Implicit rationing of nursing care, *MSV* Maximum shared variance, *NPE* Nursing practice environment

Bold diagonals in parentheses represent the square root of AVE, while italicized values above diagonal elements are the HTMT ratios

*** means *p* < 0.001

Discriminant validity was evaluated by two methods. First, Fornell and Larcker’s method recommended that the square roots of the AVE values be higher than the values of the inter-construct correlations and that the AVE be higher than the MSV [37], both of which were fulfilled. Second, Henseler et al. method required HTMT values below 0.85 [38], which were also fulfilled (Table 3). Additionally, the reliability of the study constructs was assessed through Cronbach’s alpha, which ranged from 0.91 to 0.97, surpassing the benchmark level of 0.7 [39].

### Preliminary analysis

In this study, the mean (SD) of the nursing practice environment and IRNC were 2.67 (0.54) and 1.82 (0.77), respectively, on a maximum mean score of 4. Moreover, the mean (SD) of flow and APE were 4.39 (1.14) and 2.89 (1.35), respectively, on a maximum mean score of 7. The favorable nursing practice environment was positively correlated with nurses’ flow (*r* = 0.55, *p* < 0.001, large effect size) and inversely correlated with IRNC (*r* = -0.38, *p* < 0.001, medium effect size) and APE (*r* = -0.48, *p* < 0.001, medium effect size). Additionally, nurses’ flow was inversely related to IRNC (*r* = -0.39, *p* < 0.001, medium effect size) and APE (*r* = -0.49, *p* < 0.001, medium effect size), while IRNC was positively correlated with APE (*r* = 0.37, *p* < 0.001, medium effect size; Table 3).

A stepwise multiple regression analysis was conducted to investigate the dimensions of the PES-NWI that predict flow at work. Among the five dimensions assessed, three dimensions (participation in hospital affairs, nursing foundations for quality of care, and nurse manager support) were identified as significant predictors (F = 34.84, *p* < 0.001). Furthermore, the regression coefficients indicated that nurse manager support had the most pronounced impact on nurses’ flow at work, followed by participation in hospital affairs and nursing foundations for quality of care (β = 0.277, 0.201, and 0.172, respectively; please refer to Table 4 for more details).

**Table 4:** Results of a stepwise multiple linear regression analysis predicting flow at work of the studied nurses (*N* = 231)

Predicators	B	SE (B)	β	t	P value	95% CI
						Lower/Upper
Constant	1.217	0.330		3.684	< 0.001	0.566/1.867
NMS	0.459	0.122	0.277	3.763	< 0.001	0.219/0.699
NPHA	0.378	0.142	0.201	2.663	0.008	0.098/0.658
NFCA	0.351	0.159	0.172	2.214	0.028	0.039/0.664

*NFCA* Nursing foundations for quality of care, *NMS* Nurse manager support, *NPHA* Participation in hospital affairs

F = 34.84; *p* < 0.001

### Hypotheses testing

**Fig. 2:**
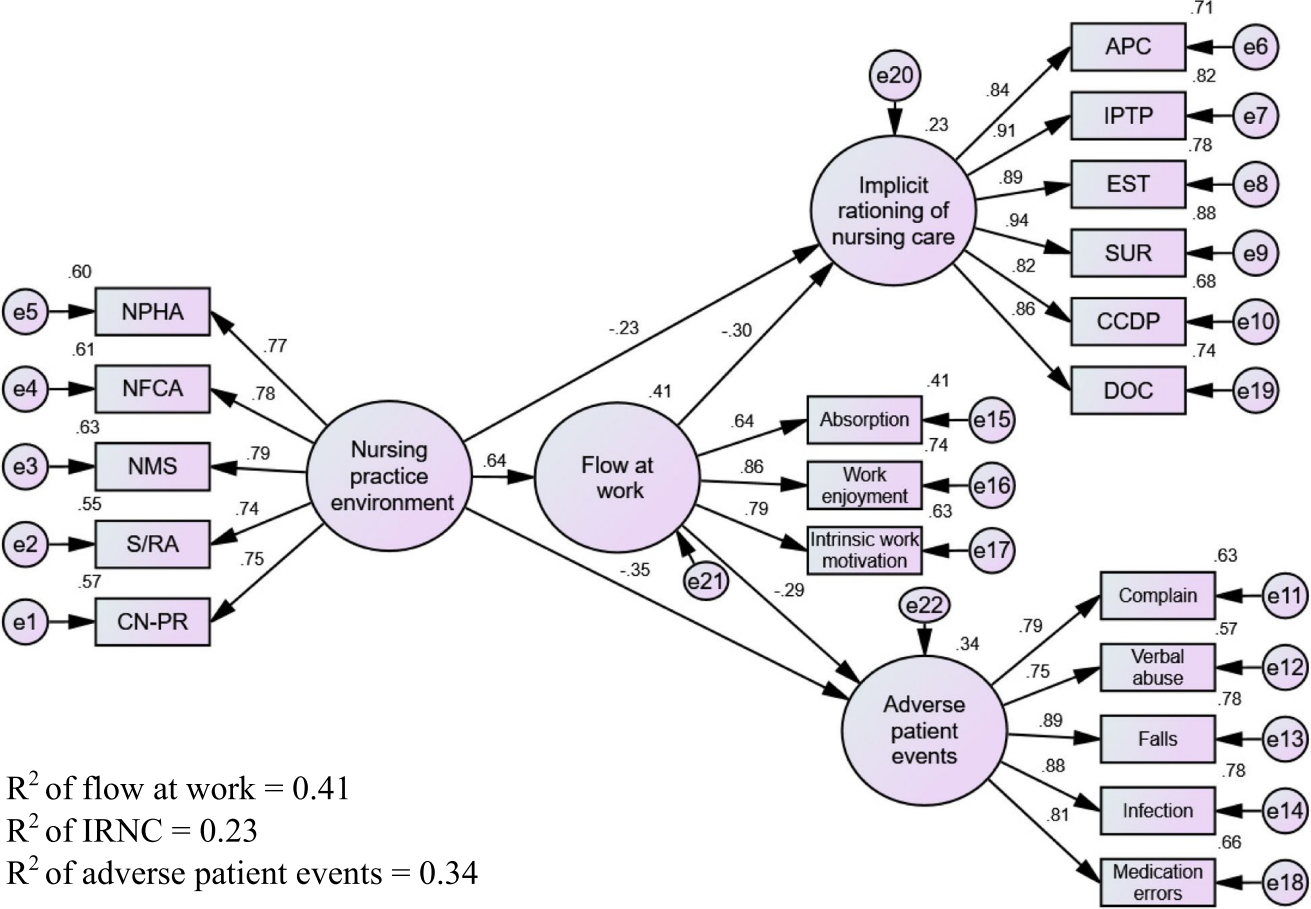
The tested model. APC: assistance with physical care; CCDP: coordination of care and discharge planning; CN-PR: collegial nurse-physician relations; DOC: documentation; EST: emotional support and teaching; IPTP: implementation of the prescribed treatment plan; NFCA: nursing foundations for quality of care; NMS: nurse manager support; NPHA: participation in hospital affairs; S/RA: staffing and resource adequacy; SUR: surveillance

The hypothesized model was tested using SEM, and the results showed a good fit (χ2 = 324.29, df = 147, χ2/df = 2.21, IFI = 0.95, TLI = 0.94, CFI = 0.95, RMSEA = 0.072). The favorable nursing practice environment significantly and positively predicted nurses’ flow (β = 0.64, *p* < 0.001) and inversely predicted IRNC (β = -0.23, *p* = 0.014) and APE (β = -0.35, *p* < 0 0.001), supporting H1, H2, and H3, respectively. Moreover, nurses’ flow significantly and inversely predicted IRNC (β = -0.30, *p* = 0.002) and APE (β = -0.29, *p* = 0.002), confirming H4 and H5, respectively. Furthermore, the 5000 bootstrap results demonstrated a significant indirect effect from the favorable nursing practice environment on IRNC (β = -0.24, *p* = 0.001, 95% CI: -0.43/-0.09) and APE (β = -0.44, *p* = 0.003; 95% CI: -0.79/-0.16) through nurses’ flow, supporting H6 and H7. The model explained 41% of the variance in nurses’ flow, 23% in IRNC, and 34% in APE (Fig. 2; Table 5).

**Table 5:** Estimates from the structural analysis (*N* = 231)

Effect	β	S.E	t	*P*	BC 95% CI
					Lower/Upper
**Direct effect**					
Nursing practice environment to flow at work	0.64	0.13	7.01	< 0.001	0.52/0.75
Nursing practice environment to IRNC	-0.23	0.12	-2.47	0.014	-0.42/-0.04
Nursing practice environment to APE	-0.35	0.22	-3.79	< 0.001	-0.52/-0.18
Flow at work to IRNC	-0.30	0.83	-3.04	0.002	-0.47/-0.12
Flow at work to APE	-0.29	0.16	-3.03	0.002	-0.47/-0.09
**Indirect effect**					
Practice environment → flow → IRNC	-0.24			0.001	-0.43 /-0.09
Practice environment → flow → APE	-0.44			0.003	-0.79 /-0.16

Standardized coefficients are reported

*APE* Adverse patient events, *BC* Bias-corrected, *CI* Confidence interval, *IRNC* Implicit rationing of nursing care, *S.E* Standard error

## Discussion

This study investigated the impact of the nursing practice environment on nurses’ experience of flow at work, IRNC, and APE. Furthermore, it explored the mediating role of flow at work in the relationship between the nursing practice environment, IRNC, and APE. Overall, the study findings confirmed the hypothesized model.

### Nursing practice environment and flow at work

As hypothesized, the study findings revealed that a favorable nursing practice environment significantly affects nurses’ flow (H1). This indicates that chaotic or poor work environments could deprive nurses of flow. A healthy work environment gives nurses the energy and vigor to carry out their duties and build resources [40]. Nurses with high personal resources typically exhibit flow [13]. This finding is consistent with studies demonstrating that positive work conditions and practices stimulate flow [11, 41]. However, this result contradicts Nielsen and Cleal, who argued that job characteristics do not predict the flow. Further research is needed to settle this controversy [42].

### Nursing practice environment, IRNC, and APE

Similar to earlier reports, the study findings demonstrated that a favorable nursing practice environment is inversely related to IRNC (H2) and APE (H3) [10, 15]. These results confirmed that building a healthy work environment catalyzes nurses to deliver effective and safe nursing care [9]. Meeting patient nursing care and ensuring patient safety are ongoing challenges for nursing practitioners and healthcare settings worldwide [7, 15]. The findings of this study can guide nurse managers to meet this challenge by promoting a supportive environment.

### Flow at work, IRNC, and APE

Our study revealed a novel finding: nurses in flow have a significant inverse effect on IRNC (H4) and APE (H5). Flow decreases nurses’ work exhaustion [13], invigorates their consciousness, and immerses them in their daily work activities [42], which may reduce errors at work. The current findings support earlier reports that flow is associated with positive job outcomes [43] and optimal job performance [44]. To the best of our knowledge, no study has related nurses’ flow at work to IRNC and APE, thus contributing new knowledge to limited studies on nurses’ flow.

### Flow at work as a mediator

As hypothesized, the study results confirmed that flow at work plays a mediating role in linking the nursing practice environment to IRNC (H6) and APE (H7). This is a significant finding as it answers the scholarly call to determine how the nursing practice environment relates to IRNC and APE. Specifically, nurses who practice in a positive work environment experience more workflow, reducing IRNC and APE. These findings align with Donabedian’s structure-process-outcome framework [17]. Additionally, our results align with the previous finding that work characteristics could enhance individuals’ flow at work, resulting in higher job performance [23].

Finally, our results indicate that the study model accounts for 23% of the variance in IRNC. This finding suggests that the model does not capture the full spectrum of factors influencing IRNC. IRNC is influenced by a range of elements within healthcare environments. Previous studies have highlighted the importance of nurse-to-patient ratios and the hours nurses work [45], in addition to the impact of nurses’ skill mix and workload [46]. The significance of nursing staff dynamics has also been emphasized [47]. Recently, focus has shifted towards examining the role of educational levels among nursing staff [48]. In light of these findings, it becomes imperative for nursing managers to implement a comprehensive approach in clinical settings, one that incorporates these varied and significant factors, to effectively address IRNC.

### Limitations

This study has several limitations worth noting. First, the study design was cross-sectional, which limited the ability to establish causality. To identify causality, longitudinal research is necessary. Second, self-report measures were used to collect data related to variables such as IRNC and APE. Self-report measures have biases, such as social desirability bias. Although the CMV test showed no evidence of social desirability bias, it is important to acknowledge that data collected through self-report methods are inherently prone to such biases. Therefore, future studies should consider using more robust data collection methods, such as direct observation or chart review. Moreover, the data collection in this study was limited to a single individual for all variables. Thus, future studies should incorporate multi-level data sources to achieve a more comprehensive understanding of the phenomena under investigation.

Additionally, the study was conducted in five hospitals in a single city in Egypt, which might limit the generalizability of the findings to other regions or settings. To enhance the study’s results, future studies should expand the scope of data collection to include multiple regions and settings.

Finally, this study was not comprehensive enough to test other mechanisms that may explain how the nursing practice environment predicts low IRNC and APE. Future research should examine other mechanisms, such as knowledge sharing, psychological ownership, or work engagement, which might explain the relationships between the nursing practice environment, the IRNC, and the APE more comprehensively.

### Implications

This study contributes to the nursing literature. Firstly, it adds theoretical value to the existing knowledge of IRNC and APE. Secondly, the study broadens the limited previous studies on nurses’ flow at work. Thirdly, the study addresses a research gap concerning the mediating effect of nurses’ flow at work on the relationship between the nursing practice environment and IRNC and APE. Moreover, to our knowledge, this study is the first to integrate these four constructs into a single structural model.

Furthermore, the study findings have practical implications for nursing management practices. The results confirm that a favorable nursing practice environment can enhance nurses’ flow and limit IRNC and APE. Therefore, nurse administrators should cultivate a better nursing practice environment by providing adequate resources and staffing, adopting supportive leadership styles, allowing nurses to participate in hospital affairs, and supporting collegial interactions among healthcare teams. Secondly, the study demonstrates that nurses’ flow at work not only curbs IRNC and APE but also serves as a mechanism that mediates the effects of the nursing practice environment on these outcomes. Hence, nurse administrators should apply interventions to foster nurses’ flow at work to improve their well-being and reduce the negative impact of IRNC and APE.

## Conclusion

The study examined the occurrence of IRNC and APE from the lens of the nursing practice environment, using nurses’ flow at work as a mediator. The study results indicate that a favorable nursing practice environment can promote nurses’ flow at work and limit the occurrence of IRNC and APE. Additionally, the study findings support the notion that nurses’ flow at work serves as a mediator, linking the nursing practice environment to IRNC and APE.

### Supplementary Information

**Additional file 1.**

## Data Availability

The datasets generated during and analyzed during the current study are not publicly available due to confidentiality agreements, but are available upon reasonable request from the corresponding author.

## References

[1] Nantsupawat A, Poghosyan L, Wichaikhum OA, Kunaviktikul W, Fang Y, Kueakomoldej S (2022). Nurse staffing, missed care, quality of care and adverse events: a cross-sectional study. J Nurs Manag.

[2] Labrague LJ, De los Santos JAA, Tsaras K, Galabay JR, Falguera CC, Rosales RA (2020). The association of nurse caring behaviours on missed nursing care, adverse patient events and perceived quality of care: a cross-sectional study. J Nurs Manag.

[3] Chaboyer W, Harbeck E, Lee BO, Grealish L (2021). Missed nursing care: an overview of reviews. Kaohsiung J Med Sci.

[4] Jones TL (2014). Validation of the perceived implicit rationing of nursing care (PIRNCA) instrument. Nurs Forum.

[5] Asif M, Jameel A, Hussain A, Hwang J, Sahito N (2019). Linking transformational leadership with nurse-assessed adverse patient outcomes and the quality of care: assessing the role of job satisfaction and structural empowerment. Int J Environ Res Public Health.

[6] Labrague LJ, Al Sabei SD, AbuAlRub RF, Burney IA, Al Rawajfah O (2021). Authentic leadership, nurse-assessed adverse patient events and quality of care: the mediating role of nurses’ safety actions. J Nurs Manag.

[7] Maghsoud F, Rezaei M, Asgarian FS, Rassouli M (2022). Workload and quality of nursing care: the mediating role of implicit rationing of nursing care, job satisfaction and emotional exhaustion by using structural equations modeling approach. BMC Nurs.

[8] Min A, Yoon YS, Hong HC, Kim YM (2020). Association between nurses’ breaks, missed nursing care and patient safety in Korean hospitals. J Nurs Manag.

[9] Kim KJ, Yoo MS, Seo EJ (2018). Exploring the influence of nursing work environment and patient sfety culture on missed nursing care in Korea. Asian Nurs Res (Korean Soc Nurs Sci).

[10] Labrague LJ, Al Sabei S, Al Rawajfah O, AbuAlRub R, Burney I (2022). Interprofessional collaboration as a mediator in the relationship between nurse work environment, patient safety outcomes and job satisfaction among nurses. J Nurs Manag.

[11] Khan MM, Mubarik MS, Ahmed SS, Islam T, Khan E (2021). Innovation with flow at work: exploring the role of servant leadership in affecting innovative work behavior through flow at work. Leadersh Organ Dev J.

[12] Zito M, Cortese CG, Colombo L (2016). Nurses’ exhaustion: the role of flow at work between job demands and job resources. J Nurs Manag.

[13] Zito M, Emanuel F, Bertola L, Russo V, Colombo L. Passion and fow at work for the reduction of exhaustion at work in nursing staff. SAGE Open. 2022;12:21582440221095010 SAGE Publications Inc..

[14] Martínez-Zaragoza F, Benavides-Gil G, Martín-Del-Rió B, Fernández-Castro J, Ato-Garciá M, Solanes-Puchol Á (2017). Flow in nurses: a study of its relationship with health and burnout in a hospital work context. Holist Nurs Pract.

[15] Zhao Y, Ma D, Wan Z, Sun D, Li H, Sun J (2020). Associations between work environment and implicit rationing of nursing care: a systematic review. J Nurs Manag.

[16] Csikszentmihalyi M (1990). Flow: the psychology of optimal experience.

[17] Donabedian A (2003). An introduction to quality assurance in health care.

[18] Ogata Y, Sato K, Sasaki M, Fujinami K, Togari T (2022). Association between nursing practice environment and sense of coherence among staff nurses: a cross-sectional study in Japan. J Nurs Manag.

[19] Lake ET (2002). Development of the practice environment scale of the nursing work index. Res Nurs Heal.

[20] Huang X, Wang L, Dong X, Li B, Wan Q (2021). Effects of nursing work environment on work-related outcomes among psychiatric nurses: a mediating model. J Psychiatr Ment Health Nurs.

[21] Schubert M, Glass TR, Clarke SP, Schaffert-Witvliet B, De Geest S (2007). Validation of the basel extent of rationing of nursing care instrument. Nurs Res.

[22] Law M, Cooper B, Strong S, Stewart D, Rigby P, Letts L (1996). The person-environment-occupation model: a transactive approach to occupational performance. Can J Occup Ther.

[23] Demerouti E (2006). Job characteristics, flow, and performance: the moderating role of conscientiousness. J Occup Health Psychol.

[24] Bachnick S, Ausserhofer D, Baernholdt M, Simon M (2018). Patient-centered care, nurse work environment and implicit rationing of nursing care in Swiss acute care hospitals: a cross-sectional multi-center study. Int J Nurs Stud.

[25] MacCallum RC, Browne MW, Sugawara HM (1996). Power analysis and determination of sample size for covariance structure modeling. Psychol Methods.

[26] Brislin RW. Back-translation for cross-cultural research. J Cross Cult Psychol. Sage PublicationsSage CA: Thousand Oaks, CA; 1970;1:185–216. Available from: http://journals.sagepub.com/doi/10.1177/135910457000100301. Cited 2021 Jan 5.

[27] Bakker AB (2008). The work-related flow inventory: construction and initial validation of the WOLF. J Vocat Behav.

[28] Uchmanowicz I, Koltuniuk A, Mlynarska A, Lagoda K, Witczak I, Rosińczuk J (2020). Polish adaptation and validation of the Perceived Implicit Rationing of Nursing Care (PIRNCA) questionnaire: a cross-sectional validation study. BMJ Open.

[29] Laschinger HK, Leiter MP (2006). The impact of nursing work environments on patient safety outcomes: the mediating role of burnout/engagement. J Nurs Adm.

[30] Labrague LJ (2021). Influence of nurse managers’ toxic leadership behaviours on nurse-reported adverse events and quality of care. J Nurs Manag.

[31] Boamah SA, Spence Laschinger HK, Wong C, Clarke S (2018). Effect of transformational leadership on job satisfaction and patient safety outcomes. Nurs Outlook.

[32] Hair JF, Black WC, Babin BJ, Anderson RE (2019). Multivariate data analysis.

[33] Collier J (2020). Applied structural equation modeling using AMOS: basic to advanced techniques.

[34] Podsakoff PM, MacKenzie SB, Podsakoff NP (2012). Sources of method bias in social science research and recommendations on how to control it. Annu Rev Psychol.

[35] Podsakoff PM, MacKenzie SB, Lee JY, Podsakoff NP (2003). Common method biases in behavioral research: a critical review of the literature and recommended remedies. J Appl Psychol.

[36] Polit DF, Beck CT (2010). Essential of nursing research: appraising evidence for nursing practice.

[37] Fornell C, Larcker DF (1981). Evaluating structural equation models with unobservable variables and measurement error. J Mark Res.

[38] Henseler J, Ringle CM, Sarstedt M (2015). A new criterion for assessing discriminant validity in variance-based structural equation modeling. J Acad Mark Sci.

[39] Nunnally J (1978). Psychometric theory.

[40] Pan X, Mao T, Zhang J, Wang J, Su P (2017). Psychological capital mediates the association between nurses’ practice environment and work engagement among Chinese male nurses. Int J Nurs Sci.

[41] Lan J, Wong CS, Jiang C, Mao Y (2017). The effect of leadership on work-related flow: a moderated mediation model. Leadersh Organ Dev J.

[42] Nielsen K, Cleal B (2010). Predicting flow at work: investigating the activities and job characteristics that predict flow states at work. J Occup Health Psychol.

[43] Shreffler J, Huecker M (2022). Physician flow at work: examining work absorption, clinical flow, work fulfillment, and flow thieves. Workplace Health Saf.

[44] Liu W, van der Linden D, Bakker AB (2022). Strengths use and work-related flow: an experience sampling study on implications for risk taking and attentional behaviors. J Manag Psychol.

[45] Assaye AM, Wiechula R, Schultz TJ, Feo R (2022). Missed nursing care, nurse staffing levels and patient safety outcomes in low-income country acute care settings: an observational study. Int J Nurs Pract.

[46] Hegney DG, Rees CS, Osseiran-Moisson R, Breen L, Eley R, Windsor C (2019). Perceptions of nursing workloads and contributing factors, and their impact on implicit care rationing: a Queensland, Australia study. J Nurs Manag.

[47] Zhao Y, Su J, Ma D, Li H, Li Y, Zhang X (2021). The role of teamwork in the implicit rationing of nursing care: a systematic mixed-methods review. J Nurs Manag.

[48] Falk AC, Boström AM, Nymark C, von Vogelsang AC. Missed nursing care in relation to registered nurses’ level of education and self-reported evidence-based practice. Worldviews Evid Based Nurs. 2023. https://sigmapubs.onlinelibrary.wiley.com/doi/epdf/10.1111/wvn.12681. ahead of print. 10.1111/wvn.12681 37735718

