# Supplementary material for: How nursing practice environments limit implicit rationing of care and nurse-assessed adverse events: the role of flow at work
Source: BMC Nurs. 2024 Jan 3;23:19. doi: 10.1186/s12912-023-01644-8 (PMC10765756; doi:10.1186/s12912-023-01644-8)
Supplement: Supplementary file 1 — Additional file 1. [file 12912_2023_1644_MOESM1_ESM.docx]

Supporting documents

| Table S1: Unit distribution across the studied hospitals and mean differences in nursing practice environment (N = 231). | | | | | |
| --- | --- | --- | --- | --- | --- |
| Hospital | Unit | no | Percent | | |
| Hospital A | Medical | 19 | 8.2 | | |
|  | Surgical | 8 | 3.5 | | |
|  | Oncology | 13 | 5.6 | | |
|  | Intensive care | 16 | 6.9 | | |
| Hospital B | Medical | 14 | 6.1 | | |
|  | Surgical | 9 | 3.9 | | |
|  | Intensive care | 17 | 7.3 | | |
| Hospital C | Medical | 15 | 6.5 | | |
|  | Surgical | 11 | 4.8 | | |
|  | Intensive care | 16 | 6.9 | | |
| Hospital D | Medical | 16 | 6.9 | | |
|  | Surgical | 10 | 4.3 | | |
|  | Intensive care | 15 | 6.5 | | |
| Hospital E | Medical | 12 | 5.2 | | |
|  | Surgical | 8 | 3.5 | | |
|  | Oncology | 14 | 6.1 | | |
|  | Intensive care | 18 | 7.8 | | |
| Mean differences of units studied in nursing practice environment | | | | | |
| Characteristic | Category | no | Percent | Nursing practice environment. | |
|  |  |  |  | M (SD) | F (P) |
| Unit | Medical | 76 | 32.9 | 2.59 (0.50) | 0.80 (0.49) |
|  | Surgical | 46 | 19.9 | 2.75 (0.63) |  |
|  | Oncology | 27 | 11.7 | 2.65 (0.54) |  |
|  | Intensive care | 82 | 35.5 | 2.69 (0.52) |  |
| Note: Hospital B, C, and D do not have an oncology unit. F represents one-way analysis of variance. | | | | | |
